# Endoprosthetic replacement with preservation of the epiphysis for proximal tibial reconstruction after osteosarcoma resection in children: a case report

**DOI:** 10.1186/s12891-024-07651-3

**Published:** 2024-07-20

**Authors:** Sijie Gui, Wantong Xu, Zhengxiao Ouyang, Xiaoning Guo, Yi Shen, Huai Tao, Xia Chen, Dan Peng

**Affiliations:** 1 Department of Orthopedics, The Second Xiangya Hospital of Central South University, Changsha, Hunan 410011 China; 2 Department of Biochemistry and Molecular Biology, Hunan University of Chinese Medicine, Changsha, Hunan 410208 China

**Keywords:** Limb salvage, Osteosarcoma, Reconstruction, Epiphyseal preservation, Endoprosthetic replacement

## Abstract

**Background:**

Limb salvage surgery is an important method for treating malignant tumors of the bone involving the adjacent parts of the major joints in children. This technique allows for preservation of limb function, especially in the lower limb. However, the reconstruction of the proximal end of the tibia after removing the tumor mass with a rational scale to preserve the total knee joint and reduce limb length discrepancy presents a challenge.

**Case presentation:**

We present a case of osteosarcoma of the proximal tibia. After being treated with an extended tumor resection, the proximal tibia of the child was restructured using endoprosthetic replacement with epiphyseal preservation. This procedure preserves the entire articular surface and growth plate of the knee joint of the affected limb and provides a feasible alternative protocol for retaining the function and growth potential of the affected limb. The patient remained disease-free and normal limb motor function was observed during the 3.5 year follow-up since the initial surgery.

**Conclusions:**

Preservation of the epiphysis enabled our patient to perform better limb function after limb-saving surgery as a result of his undamaged knee joint and minimized limb-length discrepancy. We believe that endoprosthetic replacement with preservation of the epiphysis can provide the best strategy for reconstruction after resection of focal malignant tumors in long bones without epiphytic involvement.

## Background

Osteosarcoma (OS) is one of the most common malignant bone tumors in adolescents. With the gradual development of imaging and neoadjuvant chemotherapy, limb salvage resection can provide sufficient tumor resection in children with osteosarcoma of the tibia while preserving lower limb function [1, 2]. Therefore, limb salvage surgery has become the preferred treatment option. Compared with adult patients, adolescent patients are still in the growth and development stages, and they have very high requirements for the reconstruction of the affected limb structure and function after surgery, as well as for the subsequent growth of the affected limb. Therefore, it is important to preserve normal knee joint structure as much as possible. In some cases, the growth plate of the tibia can impede the tumour spread [3]. Therefore, for a small number of patients with osteosarcoma far from the proximal tibial metaphysis, preserving the tibial epiphysis through tumor resection surgery can effectively reconstruct joint function and reduce differences in lower limb length without affecting the patient’s prognosis. Currently, customized tumor prostheses can maintain the integrity of the knee joint and growth of the femur [4, 5]; it also has the advantages of low cost, simple surgery, and high strength compared to traditional hinge knee replacement surgery.

In this report, we present the case of a 12-year-old Chinese boy with osteosarcoma of the proximal tibia who underwent limb-salvage tumor resection, followed by endoprosthetic replacement with preservation of the epiphysis. The legal guardians of the patient acquired a full description of the protocol and agreed to submit the case data for publication.

## Case presentation

**Fig. 1:**
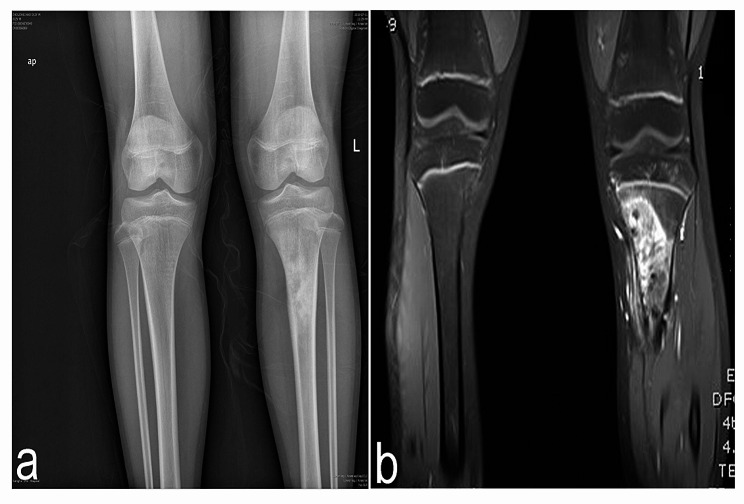
Pictures of Patient. X-ray radiograph (**a**) and magnetic resonance I maging (MRI) (**b**) of the proximal tibia, showing a lesion that did not affect the proximal epiphyseal

**Fig. 2:**
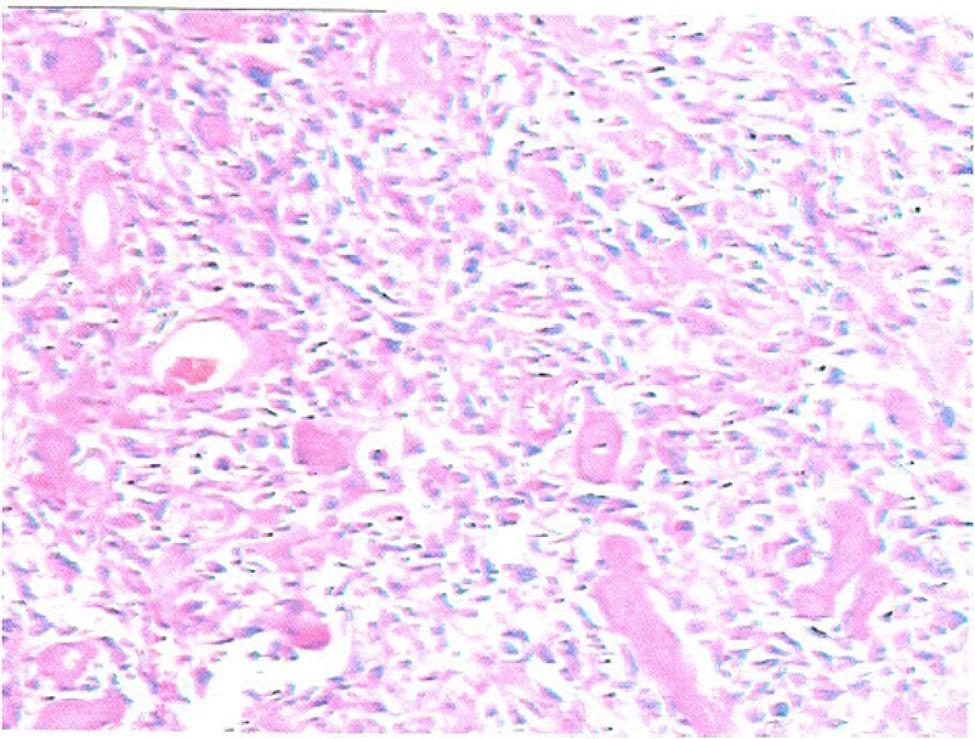
Microscopic image of the lesion with hematoxylin-eosin staining

**Fig. 3:**
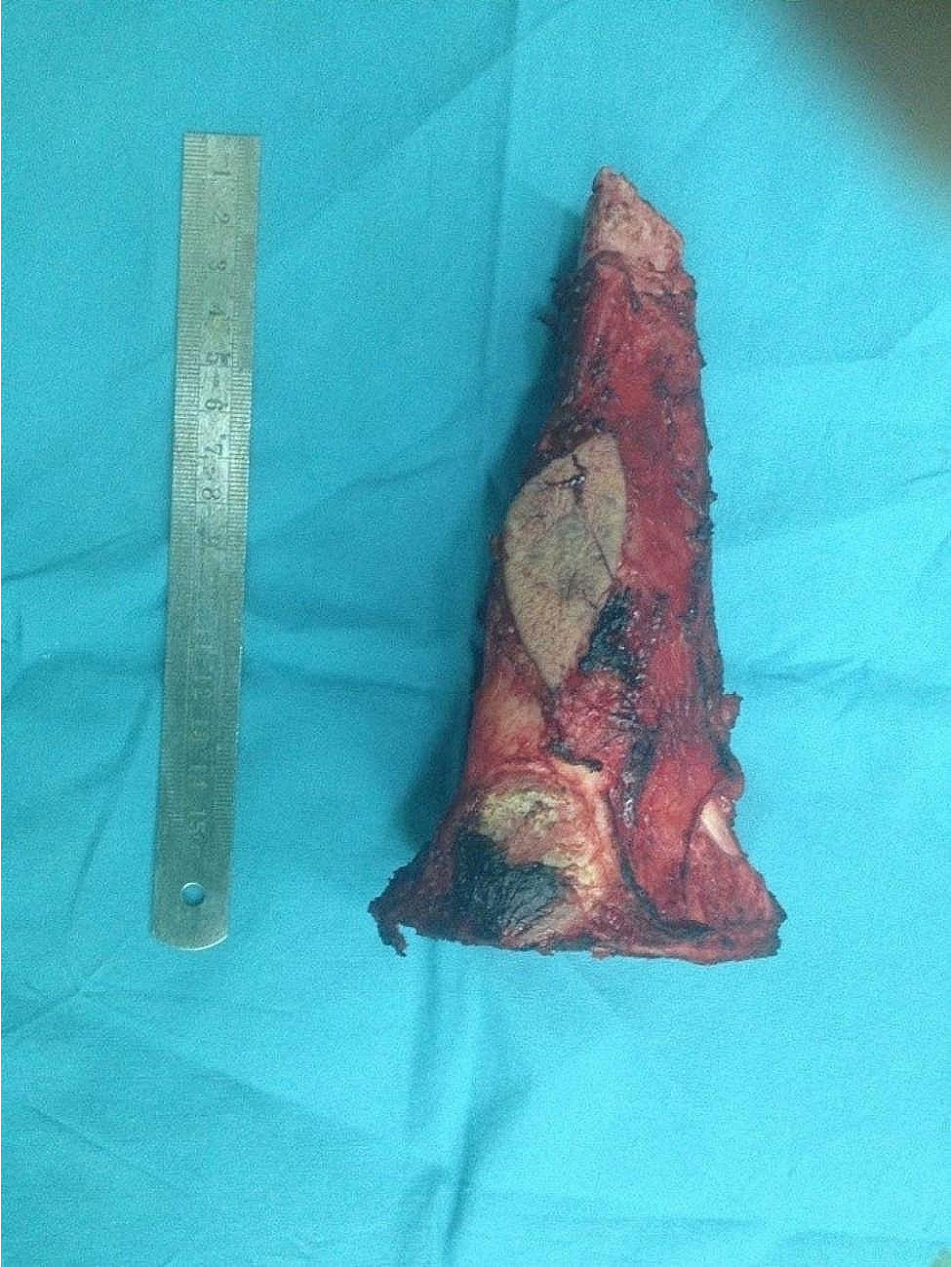
There sected tumour with a layer of normal tissue and the needle biopsy track

**Fig. 4:**
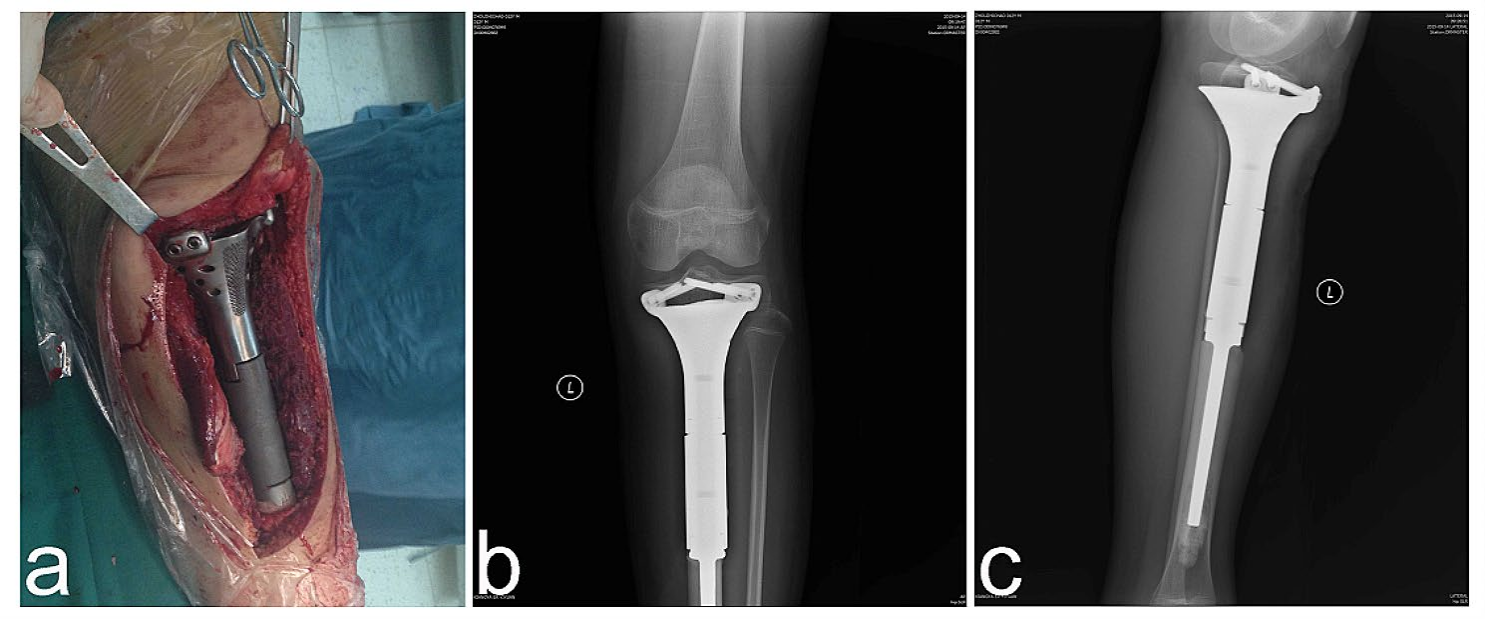
Endoprosthetic replacement for proximal tibial reconstructionin vivo. (**a**) The endoprosthesis installation was performed in vivo. Anteroposterior (**b**) and lateral (**c**) radiopraphs of the patient at the third day post-operatively following excision of the tumour and endoprosthetic replacement

**Fig. 5:**
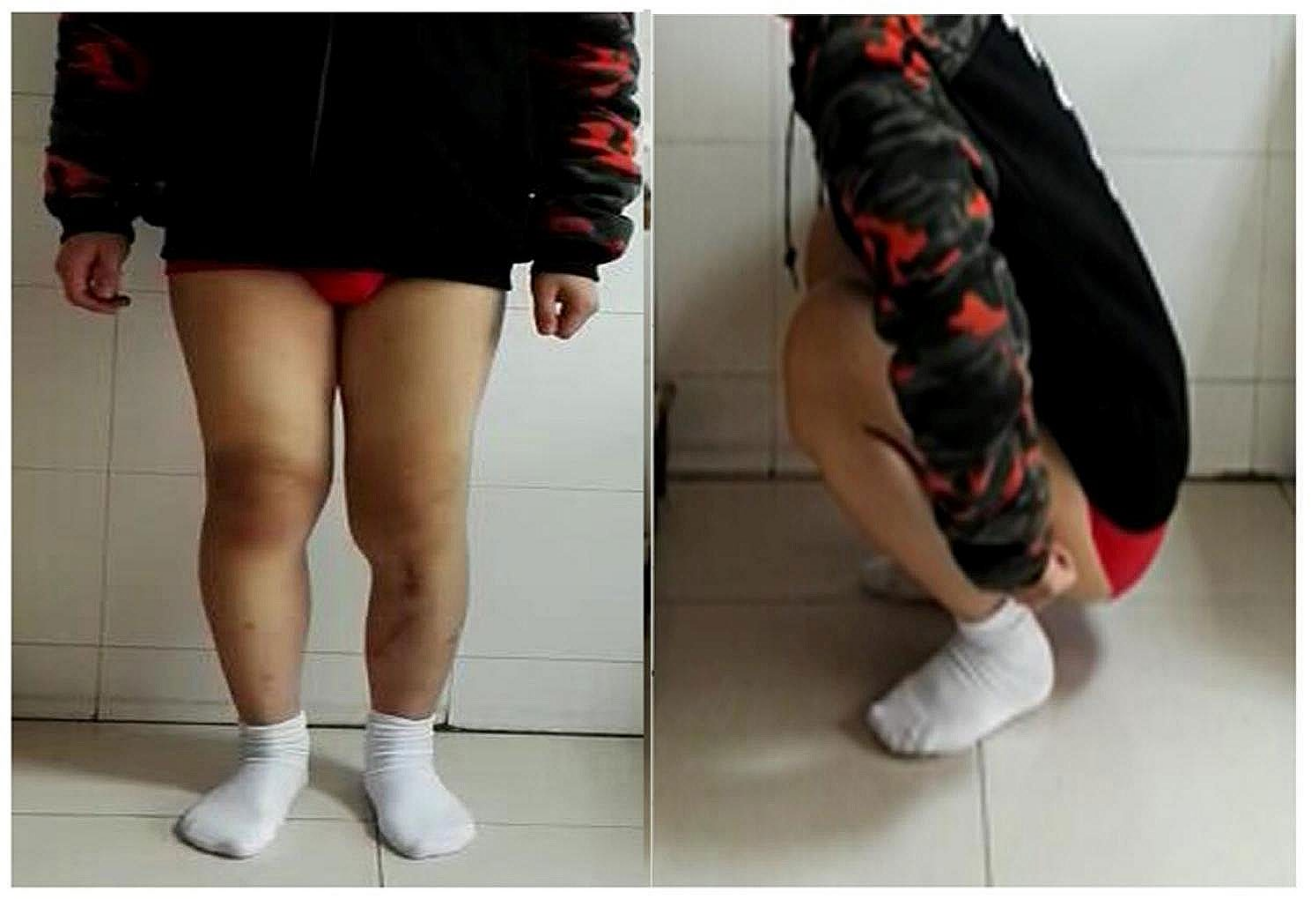
3.5 years after surgery, the affected limb was similar to the unaffected side in length, form, and function

A 12-year-old Chinese boy was brought to our hospital with complaints of soreness in the left lower leg in the previous 1 month. The inner side of the boy’s left leg was significantly swollen compared to the opposite side. Radiography findings (Fig. 1a) showed an invasive lesion in the proximal tibia with osteogenic changes and a visible periosteal reaction; however, the proximal epiphyseal plate was not invaded by the tumor tissue. In addition, as shown in Fig. 1b, MRI images of the patient’s proximal tibia showed normal medullary tissue with low signal expression being eroded by tumor tissue with high signal expression. However, the erosion range of the tumor tissue had not yet penetrated the epiphyseal plate or affected the epiphysis. Histological identification of the diagnosis was performed using a percutaneous puncture biopsy (Fig. 2). Under a microscope, a large amount of karyokinesis was observed with poor differentiation and a high cell/matrix ratio. The pathological result indicated osteosarcoma, and the Enneking surgical staging was Enneking stage IIB. Previous studies have reported that osteosarcoma should be treated with wide-margin resection and that an adequate cancer-free margin could play a pivotal role in preventing recurrence [6]. Therefore, after receiving two cycles of neoadjuvant chemotherapy (including doxorubicin, methotrexate, cyclophosphamide, vincristine, and cisplatin), soft tissue swelling and pain in the patient’s left lower limb significantly decreased, and the patient was sensitive to chemotherapy. Therefore, the patient subsequently underwent limb salvage surgery with extensive resection of left tibial osteosarcoma and prosthetic reconstruction. The level of tibial osteotomy was determined using a C-arm X-ray machine and was carefully identified. The level of the growth plate in the proximal tibia was determined as the proximal boundary of the excision, and the distal excision boundary was located in the bone shaft 3 cm away from the tumor margin. The mass, together with a layer of normal tissue around it and the track of the percutaneous biopsy, were completely removed (Fig. 3). Border imprints and gross specimens were obtained, and a pathological examination was conducted. The patient received a custom-made titanium alloy prosthesis (Beijing Chunlizhengda Medical Instruments Co., Ltd., China), after which endoprosthesis installation was performed (Fig. 4). Locking bolts were used to fix the prosthesis and the epiphysis to avoid rotation, and the epiphysis was fixed at the proximal end of the hydroxyapatite(HA)-coated prosthesis. HA coatings can promote bone lengthening and increase long-term fixation strength [7]. The ligamentum patellae were first sutured at the anchoring point at the front end of the prosthesis using non-absorbable tendon lines. Subsequently, the muscle flap obtained from the medial gastrocnemius was rotated forward to surround the frontal surface of the endoprosthesis. Finally, the medial gastrocnemius muscle flap was stitched together with the ligamentum patellae to provide stronger fixation and soft tissue coverage. During surgery, the ligament around the patient’s knee joint was preserved, which helped improve the patient’s postoperative knee joint range of motion and avoid the occurrence of limited extension. The patient began dynamic rehabilitation physiotherapy on the first day after the operation and underwent full weight-bearing exercise three weeks later. One month after surgery, the patient’s ROM reached 5°-0-120°. Three months after surgery, the patient’s ROM returned to 10°-0-145°. One month after surgery, two cycles of chemotherapy were administered according to the guidelines, totaling approximately three months (including doxorubicin, methotrexate, ifosfamide, vincristine, and cisplatin). The patient underwent routine follow-up examinations at 1, 3, and 6 months after surgery, followed by follow-up every 6 months thereafter. The patient did not show any signs of recurrence during the most recent follow-up 3.5 years after surgery. At present, the difference in length between his two limbs is very small, with his left lower limb being approximately 0.5 centimeters shorter than his right lower limb (Fig. 5). Finally, we used the Musculoskeletal Tumor Society (MSTS) scoring system to assess the patient’s recovery of limb function. At the most recent follow-up, the patient’s MSTS score was 28. Recovery of the patient’s limb motor function was satisfactory.

## Discussion

With the development of limb salvage treatments, limb salvage surgery has become increasingly preferred.

In this case, the patient was a 12 year old boy in a critical period of growth and development. Therefore, maintaining the normal biomechanical structure of the knee joint as much as possible, retaining sufficient normal bone tissue to ensure mechanical strength, and retaining normal distal femoral growth plates of the affected limb could effectively improve limb function, accelerate limb recovery, and reduce future differences in limb length. Meanwhile, based on the patient’s MRI, we found that the growth plate at the proximal tibia had not invaded. The open physis of children is a type of cartilage tissue that can hinder the spread of tumors [3, 8]. Previous studies have suggested that tumor tissue that is close to but not in contact with the epiphyseal plate can preserve the epiphyseal plate [9]. Kiss et al. [10] reported that radical excision of malignant tumors adjacent to the physeal plate with preservation of the proximal tibial epiphysis produced better limb function for the patient and did not increase the ratio of local recurrence or mortality rate in patients followed up for 4.9 years on average. Takeuchi et al. [11] also found that transverse growth impairment and collapse of the epiphysis were absent in children who underwent epiphysiological preservation surgery for osteosarcoma around the knee joint. Moreover, the radical excision of malignant tumors adjacent to the physeal plate with preservation of the proximal tibial epiphysis can preserve the complete joint surface and reduce damage to the ligaments and soft tissues around the knee joint. This can increase knee joint stability after surgery. Patients can undergo early rehabilitation exercises to restore knee joint ROM to a normal range to prevent extension lag. Therefore, we performed epiphyseal preservation resection from the proximal tibial growth plate, which can preserve sufficient mechanical strength of the epiphysis and intact the knee joint structure without affecting patient prognosis. Compared with traditional surgery, our surgical method greatly improved the functional activity of the affected limb after surgery, preserved the growth potential of the affected limb, and reduced the difference in length between the two lower limbs.

In this case, after extensive resection of the left proximal tibial osteosarcoma, a large bone defect of approximately 16 cm appeared in the tibia. Currently, the reconstruction of the proximal end of the tibia to restore a functional joint and reduce the length discrepancy of the lower legs in juveniles after resection of the bone is difficult [4, 12]. Current reconstruction strategies for proximal tibial segmental lesions include vascularized or non-vascularized autografts [13–15], allografts [16], segmental bone transport [17], prosthetic replacement, tumor segment inactivation and replantation. Autografts, allografts, and segmental bone transport prevent early weight bearing and pose a risk of disease transmission. Moreover, fixation after transplantation is influenced by adjuvant chemotherapy, which suppresses bone healing. For vascularized or non-vascularized autograft transplantation, factors such as limited graft resources, unexpected morbid donor tissue, and mismatch between the graft and defect hinder successful reconstruction [18]. Previously, prospective cohort studies have reported a considerably high morbidity of complications in reconstructions using vascularized fibular free flaps [19]. For allograft transplants, the disadvantages of reconstruction are related to high rates of fracture, nonunion, and infection following intercalary replacement [20, 21]. This suggests that children are more likely to fall victim to such complications after the fibula autograft. Segmental bone transport has drawbacks such as malunion at the anchor point and infection around the internal fixator. Ferchaud et al. [22] assessed the treatment effect of segmental bone transport using a unilateral external fixation device for several patients with femoral or tibial defects and observed re-ascending of the transported segment after external fixatorablation in some of them, thereby giving rise to a second external fixation operation and resumption of the transported segment. Patients with tumor inactivation and replantation are usually prone to complications, such as nonunion of the osteotomy, bone resorption, fracture, and infection. In summary, we believe that the preferred method of bone reconstruction in this patient was prosthetic joint replacement. As school-aged children, patients have a high demand for early recovery and mobility. Prosthetic replacement is the most common method used to reconstruct large bony defects in patients with knee osteosarcoma. The advantages of this procedure include its technical simplicity, immediate postoperative recovery of mechanical stability, early functional recovery, and maintenance of normal joint biomechanics. Therefore, for this patient, after neoadjuvant chemotherapy, prosthetic joint replacement surgery had the fastest early functional recovery, the best biomechanical structure, and fewer complications.

Customized tumor prostheses are preferred for joint replacement surgery in adolescents with preserved epiphyses. Currently, various prostheses are available, including tumor total knee prostheses [23], total proximal tibia reconstruction, expandable prostheses, and custom tumor prostheses. A major limitation of the total knee prosthesis is that it eliminates the unaffected distal femoral physis, which contributes to approximately 40% of the limb’s growth [24]. An expandable prosthesis was developed to resolve this problem. However, the expandable prostheses are expensive. In addition, invasive/noninvasive expandable prosthesis placement requires multiple operations and general anesthesia [25] and has some complications [26]. The customized tumor prosthesis not only reduces the length discrepancy of the lower leg but also has the advantages of low price, simple installment and high strength. Therefore, we believe that a customized tumor prosthesis may have been a favorable choice for this patient. Lu et al. [27] and Wong et al. [28] achieved remarkable curative effects using special tumor prostheses to repair tumor bone defects in patients. In addition, considering the risk of internal fixation loosening and infection during prosthetic replacement surgery [29], we made relevant improvements to the customized tumor prostheses. Given that aseptic loosening around the proximal prosthesis is more likely to occur than that around the distal end [30], the customized tumor prosthesis we used was incorporated into a hydroxyapatite (HA) collar. The proximal porous surface permits early cancellous bony ingrowth to obtain immediate stability, enhancing long-term fixation and reducing the infection rate caused by poor integration of the prosthesis and bone [7]. However, it is difficult to provide sufficient soft tissue coverage using traditional surgical methods. The adjacent muscle flap made of medial gastrocnemius was obtained to wrap around the anterior surface of the endoprosthesis of this patient so as to resist infection. Simultaneously, considering the postoperative knee range of the patient, we kept the collateral ligament above the proximal epiphysis of the tibia as much as possible, and preserved the medial and lateral collateral ligaments and the termination points of patellar ligaments as much as possible on the residual bone of the affected limb to help maintain knee joint balance. The patient did not experience any complications such as loosening or infection during the 3.5 year follow-up period. Therefore, choosing a customized tumor prosthesis that matches the preserved epiphysis can effectively improve limb function and early recovery, maintain a normal biomechanical structure, prevent loosening and infection, and provide a good prognosis for tibial reconstruction.

Endoprosthetic replacement with preservation of the epiphysis requires more detailed preoperative planning to ensure the accuracy of the prosthesis design and implantation. Postoperatively, problems such as uneven or excessive growth of the epiphyseal plates may occur, resulting in unequal limb lengths and changes in knee joint force lines. Although endoprosthetic replacement with preservation of the epiphysis preserves the normal joint surface and most of the surrounding ligaments of the knee joint, long-term and complex rehabilitation exercises are required after surgery.

## Conclusions

Limb salvage surgery is an important treatment for osteosarcomas adjacent to the knee joint in children and adolescents. Internal prosthesis replacement surgery that preserves the epiphysis can reconstruct the proximal tibia of children and adolescents; thus, the entire joint surface and growth plate of the knee joint of the affected limb are preserved while also preserving the function and growth potential of the affected limb. After the resection of focal malignant bone tumors in the long bone without invasion of the epiphysis, endoprosthetic replacement with preservation of the epiphysis can provide the best strategy for functional reconstruction of the affected limb.

## Data Availability

No datasets were generated or analysed during the current study.

## Abbreviations

MRI: Magnetic Resonance Imaging
MSTS: Musculoskeletal Tumor Society
