# Correction: Endoprosthetic replacement with preservation of the epiphysis for proximal tibial reconstruction after osteosarcoma resection in children: a case report

**DOI:** 10.1186/s12891-024-07844-w

**Published:** 2024-09-16

**Authors:** Sijie Gui, Wantong Xu, Zhengxiao Ouyang, Xiaoning Guo, Yi Shen, Huai Tao, Xia Chen, Dan Peng

**Affiliations:** 1 Department of Orthopedics, The Second Xiangya Hospital of Central South University, Changsha, Hunan 410011 China; 2 Department of Biochemistry and Molecular Biology, Hunan University of Chinese Medicine, Changsha, Hunan 410208 China

**Correction: BMC Musculoskelet Disord 25**,** 567 (2024)**

10.1186/s12891-024-07651-3

Following publication of the original article [1], the authors corrected the given name of the 2nd author to “Wantong” instead of “Wangtong”.

The author group has been updated above and the original article [1] has been corrected.
